# Agricultural Activities of a Meadow Eliminated Plant Litter from the Periphery of a Farmland in Inner Mongolia, China

**DOI:** 10.1371/journal.pone.0135077

**Published:** 2015-08-04

**Authors:** Kiyokazu Kawada, Wuyunna Borjigin, Toru Nakamura

**Affiliations:** 1 Faculty of Life and Environmental Sciences, University of Tsukuba, Ibaraki, Japan; 2 College of Environment and Resources, Dalian Nationalities University, Dalian, Liaoning, P. R. China; Swiss Federal Research Institute WSL, SWITZERLAND

## Abstract

The purpose of our investigation was to clarify the effects of agriculture on the process of loss of litter at the periphery of a farmland. This study revealed the generation process of an ecologically unusual phenomenon that is observed around cropland in semi-arid regions. We hypothesized that the vegetation around a farmland cannot supply plant litter to the ground surface because the ecological structure has been changed by agricultural activities. The study was conducted at Xilingol steppe, Xilingol League, Inner Mongolia Autonomous Region, China. Four study lines were established from the edge of an arable field to the surrounding meadow and parallel to the wind direction during the strong wind season. Key measurement for each line was set at the border between the farmland and steppe. Four study sites were set at intervals along each line. Plant litter, soil particle size distribution, plant species composition, plant volume, and species diversity were investigated. Despite using the same mowing method at the meadows of all study sites, the litter at the only periphery of the farmland completely disappeared. Soil particle size distribution in steppe, which was adjacent to the farmland, was similar to that of the farmland. Plant community structure at the periphery of the farmland was different from that of the far side from the farmland. This implies that soil scattered from the farmland affected the species composition of the steppe. Consequently, the change in plant community structure induced litter loss because of mowing. We concluded that plant litter was lost near the farmland because of the combined effects of farming and mowing. The results support our hypothesis that the vegetation around a farmland cannot supply plant litter because the ecological structure has been changed by agricultural activities.

## Introduction

Massive tilling generates artificially bare land that is considered a virtual desert for wildlife [1]. A notable environmental change because of steppe tilling is the increase in erosion rate [2]. The eroded soil sediment from a farmland has a high probability of affecting the periphery of the farmland. The impact of agricultural activity on this periphery has the potential to change the ecological community structure [3–5]. However, the effect on plant litter accumulation on agriculture remains unclear. Plant litter is composed of withered leaves that have accumulated on the ground. Therefore, the plant litter is closely related to the plant species composition and biomass. We predict that massive tilling eliminates litter at the periphery of the farmland, thus we examined the effect of agricultural activities on litter loss at the periphery of a farmland.

Several functional roles of plant litter in an ecosystem have been reported [6–8]. Plant litter affects numerous factors that determine the function of the entire ecosystem [9–11]. In semi-natural steppe, steppe ecosystems have been maintained by moderate disturbances such as grazing and mowing. To understand the function of litter, several studies have been conducted on litter loss [12–15]. Plant litter removal is performed to manage species diversity because the accumulation of too much litter can have a negative effect on plant diversity [12]. The lack of litter appears to be an artificial state that does not occur naturally, although this state does exist in a steppe ecosystem. Litter loss often occurs due to fire or grazing [16]. These typical disturbances in a steppe relate to variable amounts and compositions of litter. We observed litter loss in a semi-natural steppe; however, it was not disturbed by fire or grazing. Although plants were growing, it was not possible to observe any plant litter on the soil surface, which implied that the plant litter processes in this steppe ecosystem had disappeared. The process of elimination, which is different from known processes, may be related to the lost litter. However, this state was not explained because there was no clear knowledge about the process of plant litter loss. To understand the steppe ecosystem, it is necessary to clarify the process of litter loss.

Litter disappearance in semi-natural grassland was thought to be related to farmland activities because the phenomenon is only observed around the farmland, and there are no other large disturbance factors. However, because the boundary between the agricultural land and the semi-natural grassland is clear, the act of tilling is not directly involved. We considered whether indirect disturbances by farming activities were occurring, and whether the indirect effects are due to several processes. We predict that the farmland affects the environment of the periphery land, and that the plants respond to the changed environment. We aimed to understand the extent by which tilling affects litter loss in a meadow by examining changes in the environment and plant communities.

We hypothesized that the vegetation around a farmland cannot supply plant litter because an ecological structure is changed by agricultural activities. An ecological structure is composed by species composition and habitat environment. There are two steps in the process of litter loss as follows: (1) a change in the habitat environment by the deposition of sand sediment from the farmland, (2) a change in plant community at the periphery of the farmland. We focused on four factors: soil particle size distribution, species composition, plant volume, and species diversity. These characteristics denote the effects of farming on steppe ecosystems. The purpose of our investigation was to clarify the process of plant litter loss at the periphery of a farmland.

## Materials and Methods

### Study area

The Xilingol steppe in Xilingol League, Inner Mongolia Autonomous Region, China is a semi-arid region with a continental temperate steppe climate. According to data collected from 1970 to 2007 at a Baiyinxile farm in the Inner Mongolian Ecosystem Research Station of the Chinese Academy of Sciences, the annual mean temperature is 0.4°C and the lowest and highest monthly mean temperatures are −21.4°C in January and 19.0°C in July, respectively. The total annual precipitation is 337 mm and the maximum rainfall occurs between May and August [1]. This is due to the summer monsoon, and this rainfall is important for plant growth. Because of the Mongolian high-pressure current, Inner Mongolia is a region characterized by strong winds that are both arid and cold. The mean annual wind velocity is 3.7 m/s, and for 71 days, a wind velocity of over 17 m/s was recorded [17]. The main wind direction is from northwest to southeast. It causes soil erosion from early March to May in Inner Mongolia. In particular, the soil becomes dry in spring, which promotes wind erosion.

The study area was a meadow steppe near Mt. Gason (43°30′ latitude, 116°49′ longitude, altitude 1561 m) in Xilingol League, Inner Mongolia Autonomous Region, China. The area is approximately 80 km southeast of Xilinhot (capital of Xilingol League) on National Highway 303. There were no windbreaks (e.g., planted trees) at our study area. The study area is classified as a typical steppe and contains a meadow steppe biome with large arable fields. The study area has been used as a common land by people in the neighboring village. Field survey was conducted after obtaining permission in the neighboring village. It was used as a meadow, which was not used for grazing animals, because farmers with livestock use meadow steppes to reserve feed over the winter season. The area was mowed either manually or with machinery in August. In this region, immigrants from other parts of China have started to plow the steppe [18], and wheat and colza were cultivated on a farmland near the study area. According to farmers working in the arable fields, the area has been cultivated for over 40 years, with crop rotation in place and fields lying fallow every other year. There was no history of fire. We could not clarify the exact application rate of fertilizer or pesticide to the arable fields.

### Sample design and data collection

The present investigation was conducted from August 18^th^ to August 23^rd^, 2003. Four study lines (A, B, C, and D) of approximately 2 km each were established in the southeast direction from the edge of an arable field. The line direction was parallel to the wind direction during the strong wind season. Key measurements at each line were conducted at the border between the farmland and steppe. Four study sites (1, 2, 3, and 4) were set at intervals (between 300 and 1000 m) along each line, with site 1 being closest to the farmland. Distance between the steppe and farmland increases as the site number increases. At each study site, 6 study plots with a size of 1 m^2^ were set randomly.

Plant litter was collected from 2 plots at each site. The standing litter with short stems that remained after mowing was ignored. After being air-dried for approximately 3 days, the litter weight was measured. All plant species at each study plot were identified using The Key to plants at Xilin River Basin, Inner Mongolia [19]. Taxonomic nomenclature was then followed using the Flora of Inner Mongolia [20–24]. Protected species were not observed in this study. The coverage for each species was estimated using Penfound and Howard’s coverage classes [25], which were defined as follows: “+”: <1%; “1′”: 1%–5%; “1”: 5%–25%; “2”: 25%–50%; “3”: 50%–75%; and “4”: 75%–100%. The height of the tallest plant was measured for each species in all plots. The volume of plants in each plot was estimated using the *v*-value. In a previous study, the aboveground biomass of a plant community was recorded using an estimate of the *v*-value [26]. Plant volume (*v*, cm^3^) was calculated by multiplying the coverage (*c*, cm^2^) by the maximum plant height (*h*, cm) for species *i* and summing the results for all species in a plot. To calculate the volume of plants, Penfound and Howard’s coverage classes were converted to areas as follows: “+”: 50 cm^2^; “1′”: 300 cm^2^; “1”: 1600 cm^2^; “2”: 3800 cm^2^; “3”: 6300 cm^2^; and “4”: 8800 cm^2^. The volume of plants on each plot was thus calculated using the formula as follow:

$$v\;=\;\sum_{i\;=\;1}^{s}\left(ci\times hi\right)$$

Here *i* means species number of each plot and *s* denotes species richness of each plot. *ci* and *hi* denote the coverage of species *i* and the maximum plant height of species *i*.

Species diversity was analyzed using Shannon’s diversity index [27], calculated as follow:

$$H'\;=\;-\sum_{i\;=\;1}^{s}pi\log_{2}pi$$

Here *pi* denotes the relative dominance of species *i*. In this study, plant volume was used to represent dominance. Soil samples were collected from 2 plots at all sites. In addition, soil was also collected from the farmland close to site 1. Samples were collected from the soil surface (5 × 5 cm). A total of 36 soil samples (32 samples from the study sites and 4 samples from the farmland) were air-dried and sifted through a 2-mm mesh sieve to remove litter, roots, and stones. Soil particle size was measured using a laser diffraction particle size analyzer (SALD−3100; Shimadzu Co. Ltd., Kyoto, Japan).

### Data analysis

SPSS version 21 (International Business Machines Corporation (IBM), NY, USA) was used for statistical analyses. Vegetation or soil parameters were compared between the study sites using averaged values for each site to avoid pseudoreplication. The data of the six plots for each site along one line were averaged, and we used the four averaged data from the four lines for analysis. The data of plant litter were not homoscedastic. A Kruskal–Wallis test was conducted to assess the differences in plant litter among the sites. Parametric multiple comparisons (Tukey test) were conducted to assess the differences in the soil particle size of 36.8 μm, plant coverage, plant height, species richness, and species diversity among the study sites. The data of plant volume was not normally distributed, therefore nonparametric multiple comparisons (a Wilcoxon test with Bonferroni correction) were conducted to assess the differences in plant volume among the study sites. Indicator species analysis (INSPAN) [28] was used to select the indicator species at each site. Detrended correspondence analysis (DCA) [29] was performed to analyze the differences in community composition between the plots. INSPAN and DCA ordination were performed using PC–ORD for Windows version 6.08 (MjM Software Design, Gleneden Beach, OR, USA).

## Results

### Plant litter accumulation

**Fig 1:**
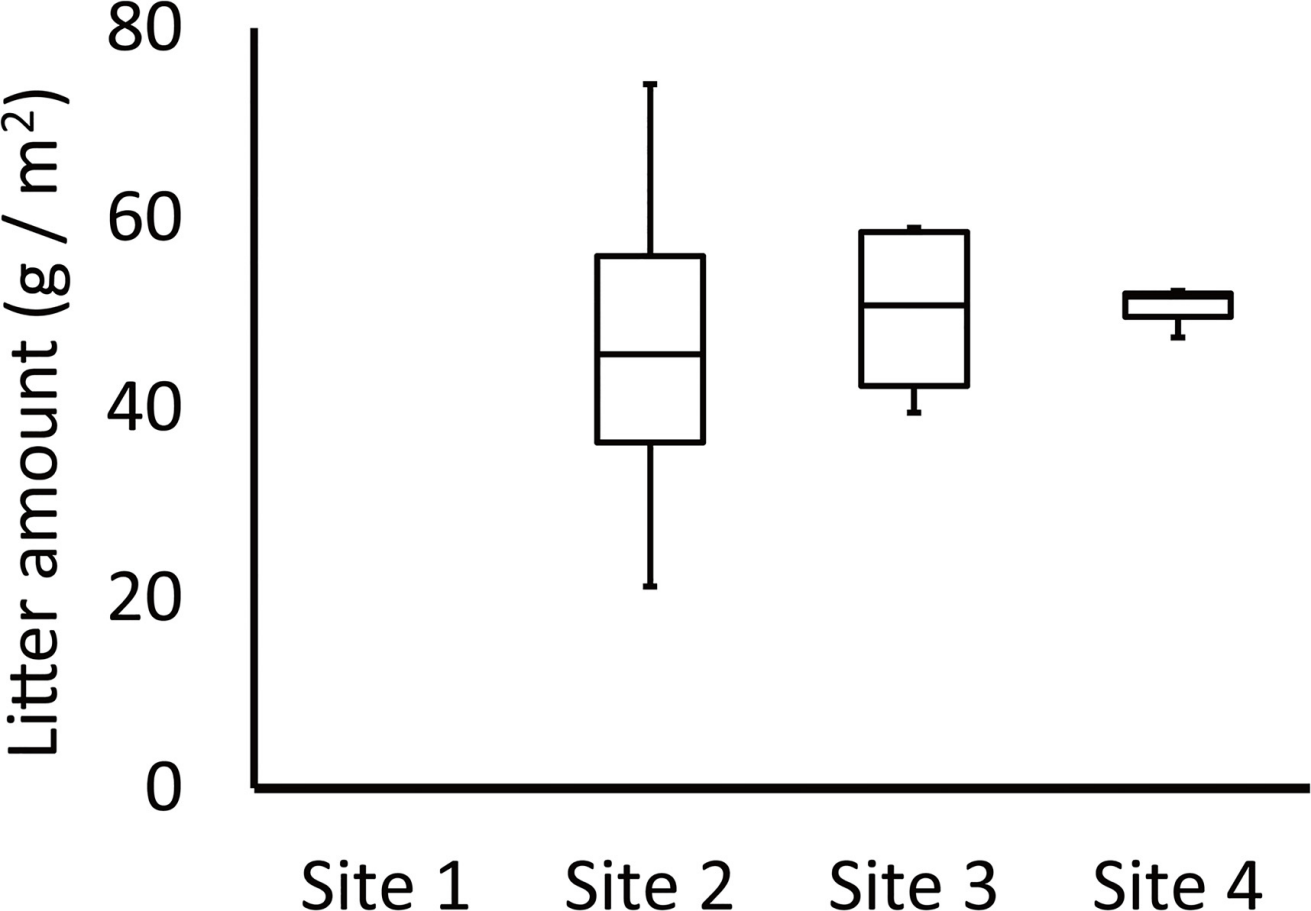
Average of above ground litter mass at the 4 study sites. Error bars denote standard error.

Plant litter accumulation was nonexistent at site 1. It was distinctly different from the other sites. Some plant litter was observed at the other sites. The average plant litter mass (average ± S.E.) at the other sites was 46.7 ± 10.9 g at site 2; 50.0 ± 5.0 g at site 3; and 48.7 ± 2.1 g at site 4 (Fig 1). The plant litter mass differed significantly among the sites (*P* < 0.05).

### Soil particle size

**Fig 2:**
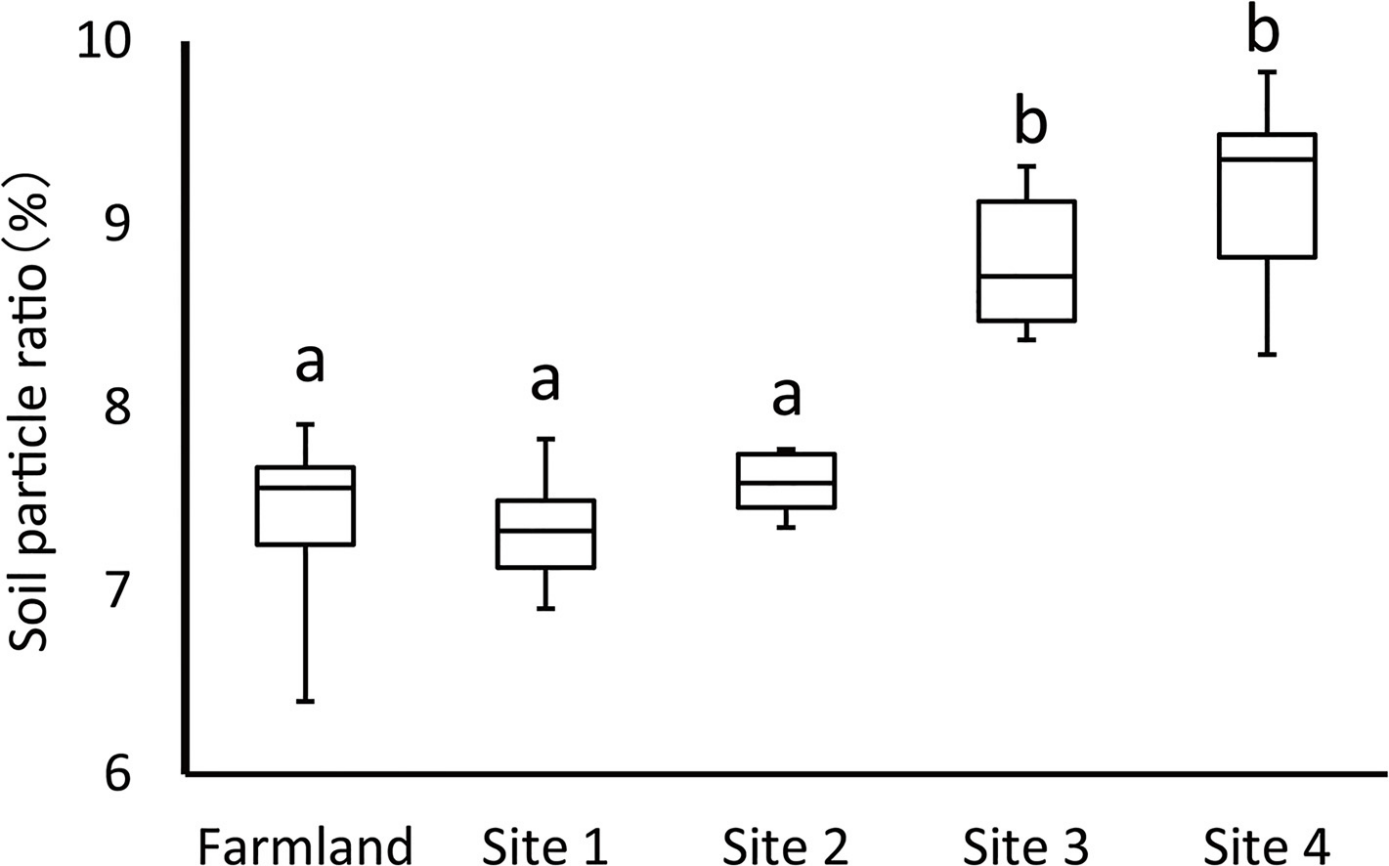
Proportions of soil particles of 36.8 μm at the four study sites and farmland.

Size distribution for soil particles of 36.8 μm was significantly different among sites (*P* < 0.01) (Fig 2). These soil particle sizes in the farmland, site 1, and site 2 were significantly lower than those in site 3 and site 4 (*P* < 0.05). Soil particle size distribution at site 1 and site 2 resembled that of the farmland, and for particles of 36.8 μm, it was not significantly different among these locations.

### Species composition and indicator species

A total of 68 species (4 species could not be identified) were identified from 96 plots (S1, S2, S3 and S4 Tables). Indicator species at each site were determined by INSPAN (S5 Table, *P* < 0.05). *Artemisia sieversiana* Ehrhart ex Willd., *Bromus inermis* Leyss., *Setaria virdis* (L.) Beauv., *Chenopodium glaucum* L. and *Chenopodium aristatum* L. were indicator species at site 1. *Leymus chinensis* (Trin.) Tzvel., *Carex korshinskyi* Kom. and *Agropyron cristatum* (L.) Gaertn. were indicator species at site 2. *Koeleria cristata* (L.) Pers., *Stipa grandis* P. Smirn., *Stellera chamaejasme* L., *Cleistogenes squarrosa* (Trin.) Keng, *Iris dichotoma* Pall., *Allium tenuissimum* L., *Cymbaria dahurica* L., *Iris ventricosa* Pall., *Filifolium sibiricum* (L.) Kitam., *Thermopsis lanceolata* R. Br., *Allium senescens* L., *Thalictrum petaloideum* L., *Heteropappus altaicus* (Willd.) Novopokr., *Adenophora stenanthina* (Ledeb.) Kitag. and *Poa subfastigiata* Trin. were indicator species at site 3. *Potentilla tanacetifolia* Willd. ex Schlecht., *Potentilla verticillaris* Steph. ex Willd., *Allium bidentatum* Fisch. Ex Prokh., *Pulsatilla turczanlnovii* Kryl. et Serg., *Artemisia eriopoda* Bunge, *Senecio kirilowii* Turcz., *Potentilla acaulis* L., *Artemisia frigida* Willd., *Scutellaria* sp., *Allium anisopodium* Ledeb., *Saposhnikovia divaricata* (Turcz.) Schischk. and *Achnatherum sibiricum* (L.) Keng. were indicator species at site 4.

### Ordination

**Fig 3:**
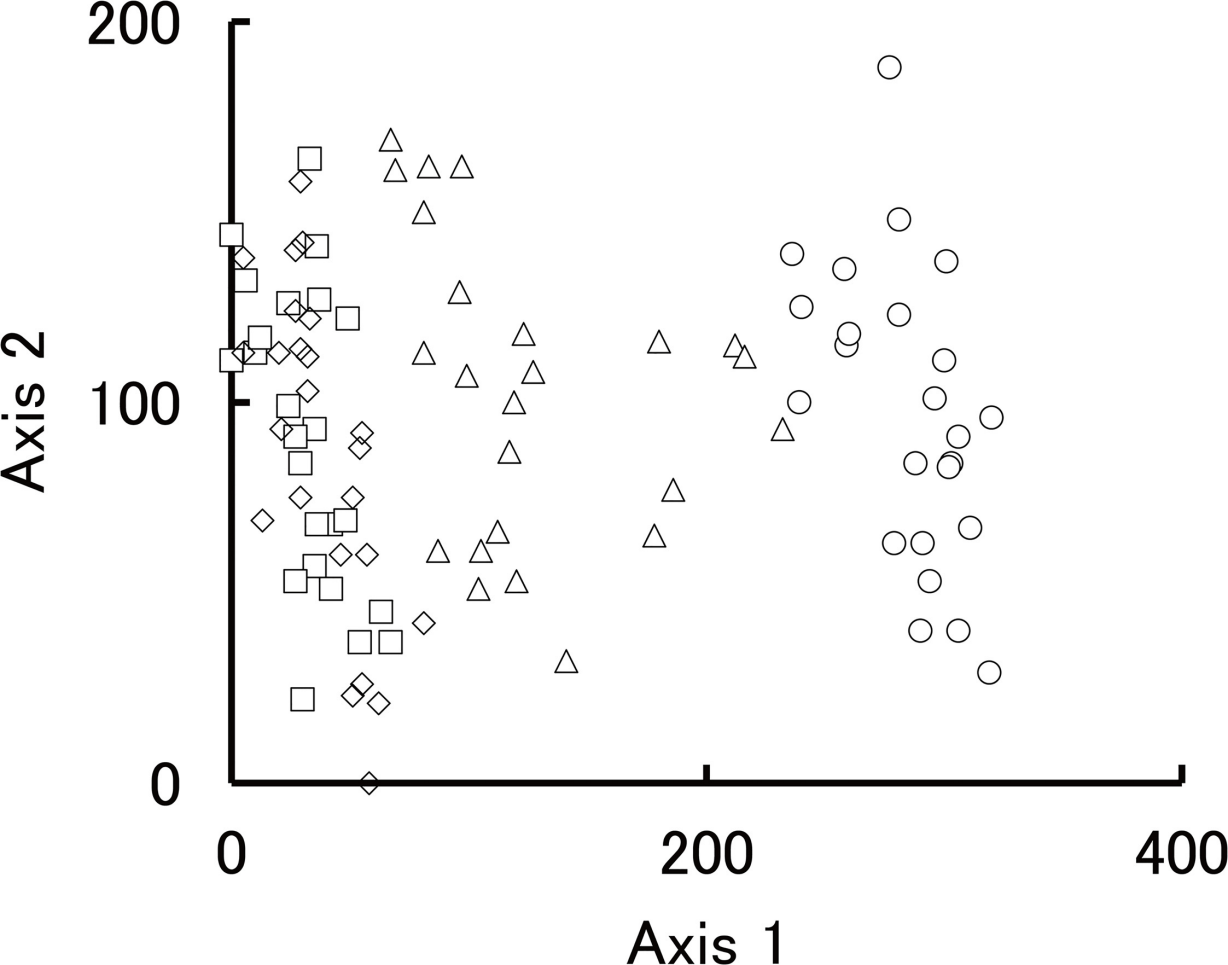
DCA ordination of the study plots. Eigenvalue of axis 1 is 0.55. Eigenvalue of axis 2 is 0.14. Symbols show the site 1 (round), site 2 (triangle), site 3 (square), and site 4 (diamond).

**Fig 4:**
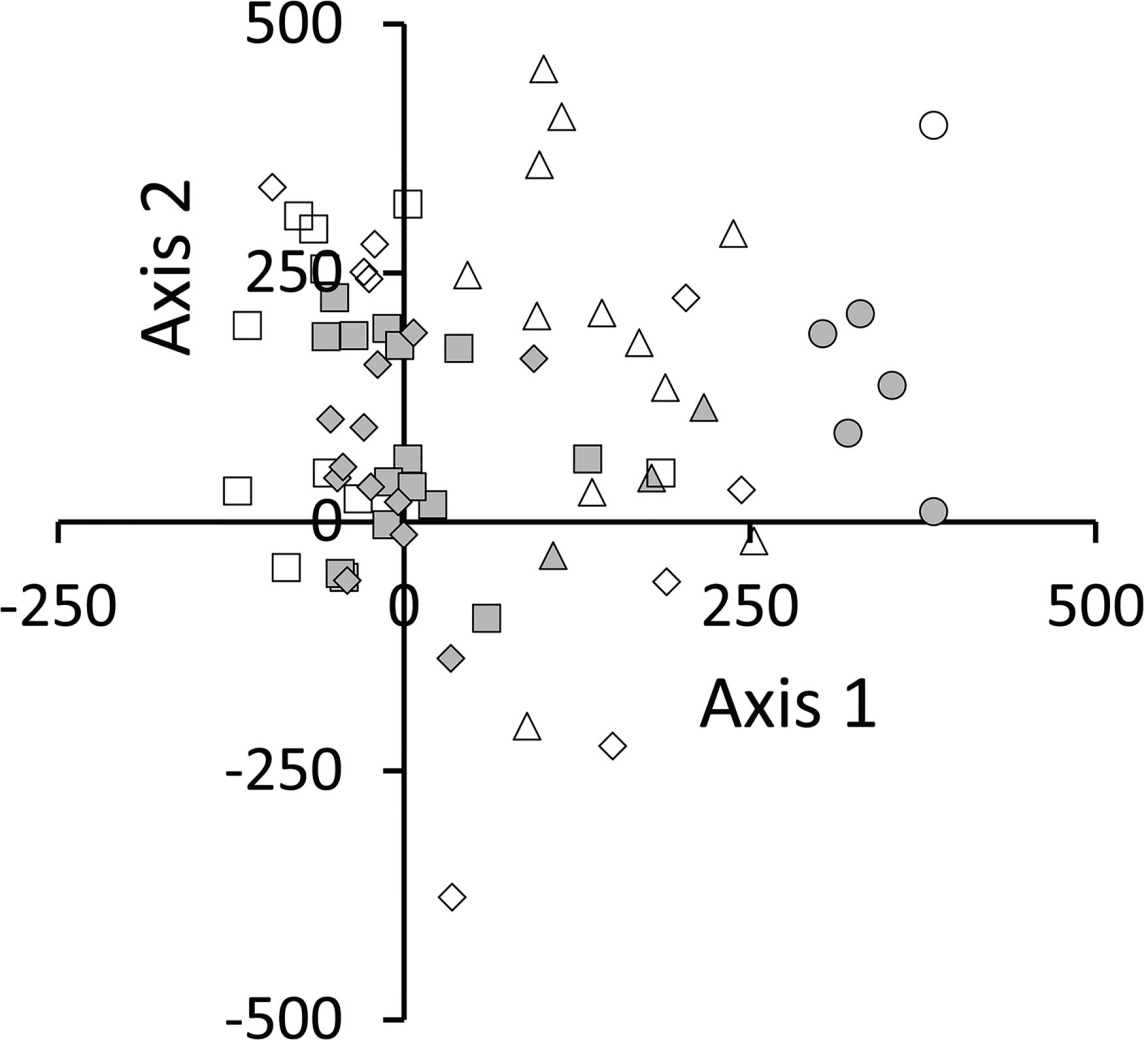
DCA ordination of the species. Eigenvalue of axis 1 is 0.55. Eigenvalue of axis 2 is 0.14. Gray-shaded symbols indicate species (INSPAN, *P* < 0.05). The vacant symbols are another appearance species that classified each group by INSPAN.

The ordination of study plots was formed at assemblage by each study site and was allocated along axis 1 (Fig 3, S6 Table). Therefore, species composition differed among the sites. Study plots located at site 1 had a high score for axis 1; however, the plots located at sites 3 and 4 had a low score for axis 1. The study plots at site 2 were located between these groups. This indicates that species composition near the farmland was different from that on the undisturbed steppe. Ordination axis 1 could be regarded as representing farming effects. The ordination of species was allocated similar to that of the ordination of study plots (Fig 4, S7 Table). The variance of indicator species scores along axis 1 was widely distributed in the ordination space similar to the variance of all species, whereas that of axis 2 was smaller than the variance of all species. The indicator species (gray-shaded symbols) could be regarded as reflecting a feature of the effects of farming (Fig 4, S5 Table).

### Vegetation characteristics

The vegetation cover at sites 1 and 2 were significantly higher than that at sites 3 and 4 (*P* < 0.05) (Table 1). The height of site 1 was significantly higher than that of the other sites (*P* < 0.05). The plant volume at site 1 was significantly higher than that at sites 3 and 4 (*P* < 0.05). The species richness at site 1 was significantly lower than that at the other sites (*P* < 0.05). In addition, the species richness at site 2 was significantly lower than that at sites 3 and 4 (*P* < 0.05). The diversity index of site 1 was significantly lower than that of the other sites (*P* < 0.05). In addition, the diversity index of site 2 was significantly lower than that of sites 3 and 4 (*P* < 0.05).

**Table 1:** Vegetation parameters at the four study sites.

	Site 1	Site 2	Site 3	Site 4
Coverage (%)	95.4 ± 1.8^a^	84.6 ± 2.8^a^	67.9 ± 4.4^b^	64.2 ± 2.6^b^
Height (cm)	39.5 ± 3.3^a^	28.9 ± 2.6^b^	23.2 ± 1.3^b^	23.3 ± 1.2^b^
Plant volume (cm^3^ × 10^−4^)	55.3 ± 10.0^a^	35.2 ± 3.4^ab^	22.0 ± 1.6^b^	19.3 ± 1.6^b^
Species richness	7.5 ± 1.0^a^	16.2 ± 2.9^b^	24.1 ± 1.0^c^	24.5 ± 1.5^c^
Species diversity (*H’*)	0.9 ± 0.2^a^	1.6 ± 0.1^b^	2.2 ± 0.1^c^	2.4 ± 0.0^c^

Means with different superscripts within a row differ (*P* < 0.05) according to multiple comparisons using a Wilcoxon test with Bonferroni correction. Numerical values indicate average ± standard error.

## Discussion

Despite using the same mowing method at all study sites, only litter at the periphery of the farmland completely disappeared (Fig 1). We considered that agricultural processes were involved in litter loss. The main cause could be the agricultural activity of tilling the steppe. Tilling causes soil to erode from the farmland and accumulate around the farmland periphery. This results in a change in species composition and a decrease in diversity at the periphery of the farmland. Consequently, the periphery of the farmland was dominated by specific species. In fact, the plant volume at site 1 is not low (Table 1). In general, litter loss is considered to be caused by a lower biomass production, however, our results do not support this because there was no litter. This is a conflicting phenomenon. On the other hand, litter sources were lost because of mowing around the farmland. If several forms of plants are growing, then plant litter of a specific life form (e.g., rosette or prostrate) can remain. However, the dominant species at site 1, *B*. *inermis*, is large and has an erect form. It is easy to harvest using a mowing machine. We considered that the litter of the dominant species was removed completely from the periphery of the farmland by mowing. These interactional processes relate to our hypothesis that the vegetation around a farmland cannot supply plant litter.

Our results showed that tilling of the meadow decreased the species number and species diversity (Table 1). A decrease in diversity is often related to agricultural activity [30]. We believe that some key environmental factors in the steppe varied at the periphery of the farmland. In this study, we focused on changes in soil particle size distribution, which is a basic soil environmental factor [31]. Soil particle size distribution at site 1 and site 2 was similar to that of the farmland (Fig 2). We consider this to be the result of soil scatter from the farmland, with soil particles accumulating in locations around the farmland. The farmland is often affected by wind erosion because the soil surface of the farmland is not covered by vegetation during the fallow period [32]. In addition, the Inner Mongolia Autonomous Region is known to experience strong winds in spring [17, 33]. Several sand scattering events resulting from strong winds occur throughout the year. Studies undertaking erosion research close to our study sites have reported that a large amount of soil erosion has occurred on the farmland [34, 35]. In locations where anti-wind erosion measures (e.g., trees planted as windbreak) are not adopted, fugitive dust from a farmland occurs routinely [36]. The accumulated soil may change the condition of steppe habitats; however, we did not clarify the chemical composition of the soil. Some fertilizers such nitrogen and phosphorus have a influence on the vegetation at the boundary of an arable field [15]. This suggests that soil scatter from the farmland possibly affected the species composition of the steppe.

Diversity decrease is often related to occupation of habitat by highly competitive species [37]. In this study, several specific species, which may have a competitive ability, were dominant in the periphery of the farmland. We observed a specific species composition near site 1 (Figs 3 and 4), suggesting that the conditions were advantageous for the growth of these particular species. At site 1, the largest volume was occupied by *B*. *inermis*. This species has a strong competitive power to extend its distribution [38]. We considered that this species extended its distribution in a short time after the accumulated soil changed the environmental conditions of its habitat. In addition, this perennial species is creating a special rhizome structure that is a physical barrier to the rhizome growth of other species [39]. It has the advantage of being able to grow even if its environment changes because of soil accumulation. We considered that the change in the species composition of the steppe is caused by soil accumulation from the farmland.

The plant volume near the farmland was higher than that in other locations far from the farmland (Table 1). This is also a result of adaptation of the species composition to site 1. An increase in biomass results in a decrease in the species diversity of a plant community, as evident from our results for species diversity (Table 1). Several reasons for the change in species composition were considered. Previous research has indicated that species composition and diversity are affected by agrochemicals [40]. In the presence of agrochemicals, the number of perennial plants, which are the dominant steppe species, decreases and the number of annual plants increases. However, many perennial plants were observed growing near the farmland, indicating that a decrease in species diversity is probably not caused by agrochemical use in this area.

We considered that the mowing of the steppe by local inhabitants was related to litter loss. Around the study site, mowing begins in the latter half of August. Local residents use the plants as feed for their livestock. Mowing is performed mainly by machine, and almost all of the aboveground biomass is removed by mowing. On steppe at the far side from the farmland, plants of various life forms construct a layer structure. These plant functional groups are important for their variety of impacts on ecosystem function [41]. After mowing, the remaining plants become litter at the end of the growing season. Individuals of low height or those with a rosette or prostrate form remain and provide plant litter after mowing. It is difficult to eliminate plant litter even if most of the aboveground parts are mowed. Nevertheless, we did not observe any litter accumulation near the farmland (Fig 3). Species diversity decreased near the farmland and created a simple community structure that was dominated by specific species (Figs 3 and 4, and Table 1). The base of stems remained standing after mowing, but were not present in the litter. We suggest that these agricultural activities result in changes in the vegetation structure and loss of litter near the farmland.

Mowing is a well-known traditional management technique for steppes and maintains high plant diversity by moderate disturbance of steppes [42, 43]. However, our results showed an inverse tendency (Table 1). We considered that the reason was duplicative actions of the agricultural system at the boundary of the farmland and grassland. Tilling of steppes accelerates the erosion of a farmland [44, 45] and changes the environment at the periphery of the farmland through the accumulation of sand sediments. Consequently, farming practices on the steppe have resulted in changes in species composition and biodiversity loss at the periphery of the farmland (Figs 3 and 4, and Table 1).

In this study, we considered a decrease in diversity as an agricultural effect at site 1 and site 2 (Table 1). We also considered that species composition was related to distance from farmland (Fig 3). However, we could not clarify the necessary strength of agricultural effects for litter loss because there was no distinct evidence to explain the relationship between species compositional change and soil sediment. In this study, at least site 1 and 2 were affected by accumulation of soil sediment (Fig 2). We were unable to clarify in detail the distance to which the effect of the farmland extended, because the distance that soil particles were transported changed according to the wind speed. Accumulation of soil sediment is also complex because it is related to topological factors. In addition, the direction of the wind is not uniform. It may be possible to estimate the range of litter loss by soil dispersal data and modeling.

## Conclusions

The combined impact of farming and mowing affected the agricultural land. The litter loss was not only caused by dispersal of sand particles from the farmland, but was also caused by mowing. We consider that it is important to understand the complex processes of litter loss: 1) dispersal of sand particles from the farmland, 2) changes in species composition, 3) increased biomass and decreased diversity, and 4) mowing. Therefore, the disappearance of litter was not caused by dispersal of sand particles from the farmland, rather it was caused by mowing.

The community structure, such as species composition, plant volume, species richness, and species diversity, was changed as a result of the accumulation of soil sediment from the farmland. In particular, the change in plant community structure resulted in a specific condition whereby the litter disappeared because of mowing. In conclusion, plant litter disappeared in the vicinity of the farmland because of the combined effect of farming and mowing. These results support our hypotheses.

Farming on steppes has an effect not only within the cultivated area but also on the original ecosystem in the surrounding area. The influence of farming on steppe ecosystems at the periphery of a farmland cannot be ignored. Our results suggest that ecosystems in arid and semi-arid regions are susceptible to environmental change as a result of farming activities.

## Supporting Information

S1 Table Species composition at Line A. The coverage for each species was estimated using Penfound and Howard’s coverage classes.
(DOCX)

S2 Table Species composition at Line B. The coverage for each species was estimated using Penfound and Howard’s coverage classes.
(DOCX)

S3 Table Species composition at Line C. The coverage for each species was estimated using Penfound and Howard’s coverage classes.
(DOCX)

S4 Table Species composition at Line D. The coverage for each species was estimated using Penfound and Howard’s coverage classes.
(DOCX)

S5 Table Results of INSPAN analysis. Indicator species at each site were determined by INSPAN (*P* < 0.05). The indicator species were marked by gray-shaded symbols (**Fig 4**).
(DOCX)

S6 Table Results of DCA analysis (Plots). Score of Ax1 and Ax2 were used to draw a scatter plot (**Fig 3**).
(DOCX)

S7 Table Results of DCA analysis (Species). Score of Ax1 and Ax2 were used to draw a scatter plot (**Fig 4**).
(DOCX)
