# Supplementary material for: Agricultural Activities of a Meadow Eliminated Plant Litter from the Periphery of a Farmland in Inner Mongolia, China
Source: PLoS One. 2015 Aug 4;10(8):e0135077. doi: 10.1371/journal.pone.0135077 (PMC4524670; doi:10.1371/journal.pone.0135077)
Supplement: S1 Table — The coverage for each species was estimated using Penfound and Howard’s coverage classes. (DOCX) [file pone.0135077.s001.docx]

**S1 Table. Species composition at Line A.** The coverage for each species was estimated using Penfound and Howard’s coverage classes.

| Site | a | a | a | a | a | a | b | b | b | b | b | b | c | c | c | c | c | c | d | d | d | d | d | d |
| --- | --- | --- | --- | --- | --- | --- | --- | --- | --- | --- | --- | --- | --- | --- | --- | --- | --- | --- | --- | --- | --- | --- | --- | --- |
| *Adenophora stenanthina* (Ledeb.) Kitag. |  |  |  |  |  | + |  |  |  |  |  | + |  |  | + |  |  |  |  |  |  |  |  |  |
| *Agropyron cristatum* (L.) Gaertn |  |  | + |  |  |  |  |  |  |  |  |  |  |  |  |  |  |  |  |  |  |  |  |  |
| *Artemisia gmelinii* Web. ex Stechm. |  |  |  |  |  |  |  |  |  |  |  |  |  |  |  |  |  |  | + |  |  |  |  | + |
| *Artemisia pubescens* Ledeb. |  |  |  |  |  |  |  |  |  |  |  |  |  |  |  |  |  |  | + | + |  |  | + |  |
| *Artemisia sieversiana* Ehrhart ex Willd. | + | + | + | + |  | + |  |  |  |  |  | + | + | + | 1 | + | + | + | 1 | + | + | + | + | + |
| *Bromus inermis* Leyss. | 3 | + | 1 | 4 | 5 | 1 | 5 | 4 | 5 | 5 | 4 | 5 | 1 | 2 | 2 | 1 | 2 | 1 | 5 | 5 | 5 | 5 | 4 | 2 |
| *Carex korshinskyi* Kom. |  |  |  | + | 1 | 1 | 1 | 1 | 1 | 1 | + | 1 |  |  |  |  |  |  |  |  | 1 |  |  |  |
| *Chenopodium aristatum* L. | + |  | + | + | + | + |  | + |  |  |  |  | + | + | + |  |  | + | 2 | 1 | 1 | + | + | 1 |
| *Chenopodium glaucum* L. |  |  | + | + | + | + |  |  |  |  |  |  |  | + | + |  | + | + | 1 | + | 1 | 1 | 1 | + |
| *Galium verum* L. |  |  |  | + |  |  |  |  |  |  |  |  |  |  |  | + | 2 | 2 | 1 |  |  |  |  | 2 |
| *Leymus chinensis* (Trin.) Tzvel. | 3 | 3 | 5 | 2 | 1 | 2 | + | 1 | + | + | 1 | 1 | 3 | 3 | 3 | 1 | 2 | 3 | + | 1 | + | + | + |  |
| *Melilotoides ruthenica* (L.) Sojak |  |  |  |  |  |  |  |  |  |  |  |  |  |  | 1 | 1 | 1 | + |  | + |  | + |  |  |
| *Potentilla bifurca* L. | + | + |  |  |  | + | + |  |  |  | + |  |  |  |  | + |  | + | 1 |  |  |  |  |  |
| *Salsola collina* Pall. |  |  |  |  |  |  |  |  |  |  |  |  |  |  |  | + |  |  | 1 |  |  |  |  |  |
| *Saposhnikovia divaricata* (Turcz.) Schischk. |  |  |  |  |  |  |  |  |  |  |  |  |  |  |  | 1 | + |  |  |  |  |  | + |  |
| *Scutellaria baicalensis* Georgi |  |  |  |  |  |  |  |  |  |  |  |  |  |  |  |  |  |  |  |  |  |  |  | 1 |
| *Serratula centauroides* L. |  |  | + |  |  | 1 | + | + | + |  | + |  |  | + | 1 | 1 |  | + |  | + | 1 | + | 2 | 2 |
| *Setaria virdis* (L.) Beauv. | + | 3 |  |  | + | 2 |  |  |  |  |  |  | 3 | 2 | 1 | 3 | 2 | 2 | 1 | 1 | 1 | 1 | 1 | 3 |
| *Thalictrum petaloideum* L. |  |  | + |  |  |  |  |  |  |  |  |  |  |  |  |  |  |  |  |  |  |  |  |  |
| *Thalictrum squarrosum* Steph. ex Willd. |  |  |  |  |  |  |  |  |  |  | + |  |  |  | 1 | 1 |  | 1 |  |  |  |  |  |  |
| *Thermopsis lanceolata* R. Br. |  |  |  |  |  |  |  |  |  |  |  |  |  | + |  |  |  | + |  |  |  |  |  |  |
| *Vicia amoena* Fisch. |  |  |  |  |  |  |  |  |  |  |  | + |  |  |  |  | + | 1 |  |  |  |  |  |  |
