# Supplementary material for: Agricultural Activities of a Meadow Eliminated Plant Litter from the Periphery of a Farmland in Inner Mongolia, China
Source: PLoS One. 2015 Aug 4;10(8):e0135077. doi: 10.1371/journal.pone.0135077 (PMC4524670; doi:10.1371/journal.pone.0135077)
Supplement: S3 Table — The coverage for each species was estimated using Penfound and Howard’s coverage classes. (DOCX) [file pone.0135077.s003.docx]

**S3 Table. Species composition at Line C.** The coverage for each species was estimated using Penfound and Howard’s coverage classes.

| Site | a | a | a | a | a | a | b | b | b | b | b | b | c | c | c | c | c | c | d | d | d | d | d | d |
| --- | --- | --- | --- | --- | --- | --- | --- | --- | --- | --- | --- | --- | --- | --- | --- | --- | --- | --- | --- | --- | --- | --- | --- | --- |
| *Achnatherum sibiricum* (L.) Keng |  |  | 1 |  |  |  |  | + |  |  |  |  |  | + | + | + | + | + |  |  |  | + |  | + |
| *Adenophora crispata* (Korsh.) Kitag. |  |  |  |  |  |  |  |  |  |  |  |  |  |  |  | + |  |  |  | + |  |  |  |  |
| *Adenophora stenanthina* (Ledeb.) Kitag. |  |  |  |  |  |  |  |  |  | + |  |  | + | + |  |  | + | + | + |  | + |  | + | + |
| *Agropyron cristatum* (L.) Gaertn | + | 1 |  |  | + |  | 1 | 1 | 2 | 2 | 2 | 2 | + | + |  | + | + |  | + | + | 2 | 1 |  | 1 |
| *Allium anisopodium* Ledeb. |  |  |  |  |  |  |  | + |  |  |  |  |  |  |  |  |  |  |  |  |  | + |  |  |
| *Allium bidentatum* Fisch. Ex Prokh. |  |  |  |  |  |  |  |  | + |  | + | + | + |  | + | + | + |  | + |  | + | + | + | + |
| *Allium condensatum* Turcz. |  |  |  |  |  |  |  | + | + | + |  | + |  |  |  |  |  |  |  |  |  |  |  | + |
| *Allium senescens* L. |  |  |  |  |  |  | + |  | + | + |  | + | + |  |  |  | + | + |  | + | + | + |  | + |
| *Allium tenuissimum* L. | + |  |  |  | + |  | + | + | + | + | + | 1 | + | + |  |  |  | + | + | + | + | + | + | + |
| *Artemisia eriopoda* Bunge |  | + | + | + | + | + |  |  | + | + | + | + |  | + |  |  | + |  | 1 | 1 | + |  |  | + |
| *Artemisia frigida* Willd. |  |  | 2 |  | 2 |  | 2 | 1 | 1 |  | 2 | 2 | + |  | 1 |  |  | + |  |  | + |  |  | 1 |
| *Artemisia pubescens* Ledeb. |  |  |  |  |  |  | + | + | + | + | + | + |  |  |  |  |  |  |  |  |  |  |  |  |
| *Astragalus melilotoides* Pall. |  |  |  |  |  |  |  |  |  |  |  |  |  | + |  |  |  |  |  |  | + |  |  |  |
| *Bupleurum scorzonerifolium* Willd. |  |  | + |  |  | + |  |  | + |  |  |  |  |  |  |  |  |  |  | + | 1 |  | + |  |
| *Carex korshinskyi* Kom. | + | + | + | + | + | 1 | + | + | 1 | 1 | + | + | 1 | + | + | + | + | + | 1 | 1 | 1 | 1 | 1 | 1 |
| *Chenopodium aristatum* L. |  |  |  |  |  |  |  | + |  |  |  |  |  |  |  |  |  |  |  |  |  |  |  |  |
| *Chenopodium glaucum* L. |  |  |  |  |  |  |  | + |  |  |  |  |  |  |  |  |  |  |  |  |  |  |  |  |
| *Cleistogenes squarrosa* (Trin.) Keng | 1 | + |  |  |  |  | + | 1 | 1 | 1 | 1 | 2 | + | + | + | + | + | + | 1 | 1 | 1 | 1 | 1 | 1 |
| *Cymbaria dahurica* L. | + | + | 1 | + | + | + | + |  | + |  |  | + | + | + | + |  | + | 1 |  | + | + | + | + | + |
| *Dianthus chinensis* L. |  |  |  | + |  |  |  |  | + |  |  |  |  |  |  |  | + |  |  |  |  |  |  |  |
| *Filifolium sibiricum* (L.) Kitam. | 1 | 1 | 1 | + | 1 | 1 | 1 |  | + |  | + |  | 2 | 2 | 2 | 3 | 2 | 3 |  | + |  |  | + |  |
| *Heteropappus altaicus* (Willd.) Novopokr. |  |  | + | + |  |  | + | 2 |  |  | + | + |  |  |  |  |  |  |  |  |  |  |  |  |
| *Iris dichotoma* Pall. | + | + |  |  | + |  | + |  | + | 1 | + | 1 | + | + | + | + | + | + | + | + | + | + | + | + |
| *Iris ventricosa* Pall. |  |  |  | + |  |  | + |  |  |  |  |  | + |  | + | + | + | + | + | + | + | + | + | + |
| *Koeleria cristata* (L.) Pers. | + | 1 | + | + | + | + | + | 1 | 1 | 2 | 1 | 1 | + |  | + | + | + | + | 1 | 1 | 1 | 1 | + | + |
| *Leontopodium leontpodioides* (Wild.) Beauv. |  |  |  |  |  |  |  | + |  |  |  |  |  |  | + | + |  | + |  | + |  |  | + |  |
| *Leymus chinensis* (Trin.) Tzvel. | + | + | + | 1 | + | + | 2 | 3 | 1 | 1 | + | + | 2 | 1 | + | + | + | + | 1 | + | 1 | + | + | + |
| *Linum perenne* L. |  |  |  |  |  |  |  |  |  |  |  |  |  |  |  |  |  |  |  |  |  |  |  | + |
| *Melilotoides ruthenica* (L.) Sojak |  |  |  |  |  |  | + | + | + | + | + |  | + | + | + | + | + | + |  | + |  | + | + |  |
| *Oxytropis myriophylla* (Pall.) DC. | + |  |  |  | + |  | + |  |  |  |  |  |  |  | + | + |  |  |  |  |  |  |  |  |
| *Poa subfastigiata* Trin. |  |  |  |  | + |  | + | + | + |  |  | + | + | + |  |  |  |  | + |  |  | + | + |  |
| *Potentilla acaulis* L. |  |  |  | + |  |  |  | 1 | 1 |  |  |  | 2 | 3 | 3 | 1 | 1 | 1 | + |  | + | 1 |  | 1 |
| *Potentilla bifurca* L. | 1 |  | + |  | + | + |  |  |  |  | + |  | + |  |  |  |  |  | + | + | + | + | + | + |
| *Potentilla parvifolia* Fisch. ap. Lehm. |  |  |  |  | + | + |  |  |  |  |  |  | + |  |  |  |  |  |  |  |  |  |  |  |
| *Potentilla tanacetifolia*Willd. ex Schlecht. |  |  | + | + | 1 | + | + | 1 | + | 1 |  |  |  | + | 1 | + | + |  | 2 | + | 1 | 1 | 2 | 3 |
| *Potentilla verticillaris* Steph. ex Willd. | 1 | + | 1 | + | + | + |  |  |  |  |  |  | + |  | + | + | + | + | 1 | + | + | + | 1 | + |
| *Pulsatilla turczaninovii* Kryl. et Serg. | + |  | + | + | + | + |  | + | + | + | + | + | + |  | + | + | + | + |  | + | 1 | 1 | 2 | 1 |
| *Sanguisorba officinalis* L. |  |  |  | 1 |  |  |  |  |  |  |  |  |  |  |  | + |  |  |  |  |  |  |  |  |
| *Saposhnikovia divaricata* (Turcz.) Schischk. | 1 | 1 |  | + | 1 | + | + | + | 2 |  | + | + |  | + | + | + |  | + | + | + | + | + |  |  |
| *Scabiosa comosa* Fisch. ex Roem. et schult. |  | 1 |  | + |  |  |  |  |  | + |  |  |  |  |  | 1 | 2 |  |  | 1 |  |  |  |  |
| *Schizonepeta multifida* (L.) Briq. | + |  |  |  |  |  |  |  |  |  |  |  |  |  | 1 | 1 | 1 | + |  |  |  |  |  | + |
| *Scutellaria baicalensis* Georgi |  |  |  |  | 1 | 1 |  |  |  | + |  |  |  |  |  |  |  |  |  |  |  |  |  |  |
| *Scutellaria scordifolia* Fisch. ex Schrank |  |  |  |  |  | 1 |  | + |  |  | + | + |  |  | + |  |  |  |  |  |  | + |  |  |
| *Scutellaria* sp. |  |  |  |  |  |  |  |  |  |  | + | + |  |  |  |  |  |  | + | + | + |  |  |  |
| *Senecio kirilovii* Turcz. ex DC. |  |  | + | 1 | + |  |  |  | + |  |  |  |  |  |  |  |  |  |  |  |  |  |  |  |
| *Serratula centauroides* L. | + | 1 | 1 | 1 | 1 | + | + | + | + | 1 | + | + | + | + |  | + | + | 1 |  | + |  | 1 | 1 | + |
| *Setaria virdis* (L.) Beauv. |  | + |  |  |  |  |  |  |  |  |  |  |  |  |  |  |  |  |  |  |  |  |  |  |
| *Silene jenissconsis* Willd. | 1 | + | + | + | + | 1 |  |  |  |  |  |  |  |  |  | + | + | + |  |  |  |  |  |  |
| *Stellera chamaejasme* L. | + | 1 | 2 | 1 | + | 1 | 1 |  |  |  |  | + | + | 1 | + | 1 | 1 | + | 2 | + |  | + | + | 1 |
| *Stipa grandis* P. Smirn. | 3 | 2 | 2 | 3 | 1 | 2 | 2 | 1 | 2 | 1 | 2 | 1 | 2 | 2 | 1 | 1 | 2 | 1 | 1 | 2 | 2 | 1 | 2 | 1 |
| *Thalictrum petaloideum* L. | + |  | + |  | + | + |  | + | + |  |  | + |  | + | + | + | + | + | + |  | + | + | + |  |
| *Thalictrum squarrosum* Steph. ex Willd. |  | + |  | 1 | + |  |  |  |  |  |  |  |  |  |  |  |  |  | 1 | 1 | 1 | 2 |  | + |
| *Thermopsis lanceolata* R. Br. |  |  | + | + | + |  |  |  |  |  |  |  | + | + | + | + | + |  |  | + |  | 1 | + | + |
| unknown sp.2 |  |  | + |  |  |  |  |  |  |  |  |  |  |  |  |  |  |  |  |  |  |  |  |  |
| *Vicia amoena* Fisch. |  |  |  |  |  |  |  |  |  |  | 1 |  |  |  |  |  |  |  |  |  |  |  |  | + |
