# Supplementary material for: Agricultural Activities of a Meadow Eliminated Plant Litter from the Periphery of a Farmland in Inner Mongolia, China
Source: PLoS One. 2015 Aug 4;10(8):e0135077. doi: 10.1371/journal.pone.0135077 (PMC4524670; doi:10.1371/journal.pone.0135077)
Supplement: S5 Table — Indicator species at each site were determined by INSPAN (P < 0.05). The indicator species were marked by gray-shaded symbols (Fig 4). (DOCX) [file pone.0135077.s005.docx]

**S5 Table. Results of INSPAN analysis.** Indicator species at each site were determined by INSPAN (*P* < 0.05). The indicator species were marked by gray-shaded symbols (**Figure 4**).

| Species | Group | Indicator Value | Mean | S. D. | P value |
| --- | --- | --- | --- | --- | --- |
| *Artemisia sieversiana* Ehrhart ex Willd. | 1 | 62.5 | 11.2 | 3.38 | 0.0002 |
| *Bromus inermis* Leyss. | 1 | 92.7 | 13.4 | 3.52 | 0.0002 |
| *Setaria virdis* (L.) Beauv. | 1 | 48.3 | 14.4 | 3.59 | 0.0002 |
| *Chenopodium glaucum* L. | 1 | 35 | 12.8 | 3.48 | 0.0004 |
| *Chenopodium aristatum* L. | 1 | 34.1 | 15.1 | 3.58 | 0.0008 |
| *Artemisia gmelinii* Web. ex Stechm. | 1 | 8.3 | 3.6 | 2.66 | 0.2372 |
| *Leymus chinensis* (Trin.) Tzvel. | 2 | 41.3 | 28 | 1.66 | 0.0002 |
| *Carex korshinskyi* Kom. | 2 | 35.4 | 25.5 | 2.04 | 0.0002 |
| *Agropyron cristatum* (L.) Gaertn | 2 | 30.3 | 20.8 | 3.13 | 0.0112 |
| *Dianthus chinensis* L. | 2 | 16.7 | 9.4 | 3.22 | 0.0674 |
| *Serratula centauroides* L. | 2 | 28.8 | 25.5 | 2.12 | 0.0928 |
| *Scutellaria scordifolia* Fisch. ex Schrank | 2 | 14 | 10.1 | 3.23 | 0.1478 |
| *Scutellaria baicalensis* Georgi | 2 | 10 | 8.1 | 3.04 | 0.1804 |
| *Clematis hexapetala* Pall. | 2 | 8.3 | 3.6 | 2.66 | 0.2388 |
| *Allium condensatum* Turcz. | 2 | 9.7 | 8.5 | 3.24 | 0.3135 |
| *Melilotoides ruthenica* (L.) Sojak | 2 | 18 | 18 | 3.42 | 0.4453 |
| *Potentilla bifurca* L. | 2 | 17 | 18.2 | 3.39 | 0.6005 |
| *Lappula redowskii* (Horn) Greene | 2 | 4.2 | 4.2 | 0.06 | 1 |
| *Polygonum divaricatum* L. | 2 | 4.2 | 4.2 | 0.06 | 1 |
| *Geranium sibricum* L. | 2 | 4.2 | 4.2 | 0.06 | 1 |
| *Rumex* sp. | 2 | 4.2 | 4.2 | 0.06 | 1 |
| *Saussurea japonica* (Thunb.) DC. | 2 | 4.2 | 4.2 | 0.06 | 1 |
| *Koeleria cristata* (L.) Pers. | 3 | 37 | 20.9 | 3.2 | 0.0002 |
| *Stipa grandis* P. Smirn. | 3 | 40.3 | 22.6 | 2.69 | 0.0002 |
| *Stellera chamaejasme* L. | 3 | 44.6 | 15.6 | 3.4 | 0.0002 |
| *Cleistogenes squarrosa* (Trin.) Keng | 3 | 43 | 17 | 3.41 | 0.0002 |
| *Iris dichotoma* Pall. | 3 | 37.4 | 18.1 | 3.26 | 0.0004 |
| *Allium tenuissimum* L. | 3 | 33.6 | 15.7 | 3.33 | 0.001 |
| *Cymbaria dahurica* L. | 3 | 30.8 | 18.1 | 3.16 | 0.0016 |
| *Iris ventricosa* Pall. | 3 | 27.1 | 12.2 | 3.46 | 0.0022 |
| *Filifolium sibiricum* (L.) Kitam. | 3 | 32 | 16.6 | 3.55 | 0.0028 |
| *Thermopsis lanceolata* R. Br. | 3 | 25 | 12 | 3.32 | 0.0056 |
| *Allium senescens* L. | 3 | 24 | 11 | 3.33 | 0.0066 |
| *Thalictrum petaloideum* L. | 3 | 26 | 16.5 | 3.22 | 0.0154 |
| *Heteropappus altaicus* (Willd.) Novopokr. | 3 | 18.2 | 7.2 | 3.18 | 0.021 |
| *Adenophora stenanthina* (Ledeb.) Kitag. | 3 | 18.8 | 9.9 | 3.31 | 0.0232 |
| *Poa subfastigiata* Trin. | 3 | 18.9 | 11.1 | 3.24 | 0.0286 |
| *Silene jenissconsis* Willd. | 3 | 17.2 | 10.6 | 3.47 | 0.0594 |
| *Oxytropis myriophylla* (Pall.) DC. | 3 | 13 | 6.6 | 2.95 | 0.0878 |
| *Bupleurum scorzonerifolium* Willd. | 3 | 13.5 | 8.2 | 3.18 | 0.1026 |
| *Leontopodium leontpodioides* (Wild.) Beauv. | 3 | 13.6 | 7.7 | 3.17 | 0.1118 |
| *Potentilla parvifolia* Fisch. ap. Lehm. | 3 | 9.4 | 4.9 | 2.69 | 0.1888 |
| *Thalictrum squarrosum* Steph. ex Willd. | 3 | 14.1 | 11.9 | 3.43 | 0.2124 |
| *Adenophora crispata* (Korsh.) Kitag. | 3 | 8.3 | 3.6 | 2.7 | 0.2492 |
| *Sanguisorba officinalis* L. | 3 | 5 | 5.1 | 2.69 | 0.6245 |
| *Linum perenne* L. | 3 | 2.1 | 3.6 | 2.68 | 1 |
| unknown sp. 2 | 3 | 4.2 | 4.2 | 0.06 | 1 |
| *Potentilla tanacetifolia*Willd. ex Schlecht. | 4 | 44.8 | 20.4 | 3.14 | 0.0002 |
| *Potentilla verticillaris* Steph. ex Willd. | 4 | 37.5 | 18.7 | 3.27 | 0.0002 |
| *Allium bidentatum* Fisch. Ex Prokh. | 4 | 35.4 | 14.8 | 3.37 | 0.0002 |
| *Pulsatilla turczaninovii* Kryl. et Serg. | 4 | 38.1 | 17.6 | 3.42 | 0.0002 |
| *Artemisia eriopoda* Bunge | 4 | 43.3 | 15.3 | 3.32 | 0.0002 |
| *Senecio kirilovii* Turcz. ex DC. | 4 | 28 | 9.8 | 3.27 | 0.0004 |
| *Potentilla acaulis* L. | 4 | 36.6 | 14.6 | 3.55 | 0.0004 |
| *Artemisia frigida* Willd. | 4 | 29.2 | 15.6 | 3.43 | 0.0016 |
| *Scutellaria* sp. | 4 | 24.1 | 8.8 | 3.21 | 0.002 |
| *Allium anisopodium* Ledeb. | 4 | 16.7 | 6.9 | 3.05 | 0.0296 |
| *Saposhnikovia divaricata* (Turcz.) Schischk. | 4 | 27.4 | 20.6 | 3.16 | 0.0372 |
| *Achnatherum sibiricum* (L.) Keng | 4 | 18.9 | 11.2 | 3.4 | 0.0388 |
| *Artemisia pubescens* Ledeb. | 4 | 14.8 | 9.2 | 3.29 | 0.0576 |
| unknown sp. 1 | 4 | 12.5 | 4.5 | 2.79 | 0.0576 |
| *Schizonepeta multifida* (L.) Briq. | 4 | 16.1 | 10.6 | 3.49 | 0.0828 |
| *Galium verum* L. | 4 | 15 | 12.5 | 3.53 | 0.2128 |
| *Scabiosa comosa* Fisch. ex Roem. et schult. | 4 | 12.1 | 11 | 3.45 | 0.2817 |
| Vicia amoena Fisch. | 4 | 10.5 | 8.9 | 3.26 | 0.2831 |
| *Astragalus melilotoides* Pall. | 4 | 8.3 | 6.5 | 3.01 | 0.4049 |
| *Salsola collina* Pall. | 4 | 6.7 | 7 | 3.07 | 0.5791 |
| *Orostachys fimbriatus* (Turcz.) Berger | 4 | 4.2 | 4.2 | 0.06 | 1 |
