# Supplementary material for: Agricultural Activities of a Meadow Eliminated Plant Litter from the Periphery of a Farmland in Inner Mongolia, China
Source: PLoS One. 2015 Aug 4;10(8):e0135077. doi: 10.1371/journal.pone.0135077 (PMC4524670; doi:10.1371/journal.pone.0135077)
Supplement: S6 Table — Score of Ax1 and Ax2 were used to draw a scatter plot (Fig 3). (DOCX) [file pone.0135077.s006.docx]

**S6 Table. Results of DCA analysis (Plots).** Score of Ax1 and Ax2 were used to draw a scatter plot (**Figure 3**).

| Line | Site | Plot | AX1 | AX2 | AX3 |
| --- | --- | --- | --- | --- | --- |
| A | 1 | 1 | 296 | 101 | 110 |
| A | 1 | 2 | 281 | 148 | 189 |
| A | 1 | 3 | 239 | 100 | 141 |
| A | 1 | 4 | 302 | 83 | 90 |
| A | 1 | 5 | 311 | 67 | 74 |
| A | 1 | 6 | 260 | 118 | 161 |
| A | 2 | 1 | 212 | 115 | 156 |
| A | 2 | 2 | 180 | 116 | 226 |
| A | 2 | 3 | 178 | 65 | 118 |
| A | 2 | 4 | 232 | 93 | 182 |
| A | 2 | 5 | 216 | 112 | 153 |
| A | 2 | 6 | 186 | 77 | 105 |
| A | 3 | 1 | 24 | 126 | 151 |
| A | 3 | 2 | 49 | 122 | 162 |
| A | 3 | 3 | 10 | 113 | 121 |
| A | 3 | 4 | 36 | 141 | 129 |
| A | 3 | 5 | 37 | 127 | 132 |
| A | 3 | 6 | 33 | 164 | 117 |
| A | 4 | 1 | 27 | 140 | 152 |
| A | 4 | 2 | 5 | 138 | 135 |
| A | 4 | 3 | 29 | 114 | 142 |
| A | 4 | 4 | 5 | 113 | 133 |
| A | 4 | 5 | 29 | 158 | 130 |
| A | 4 | 6 | 29 | 75 | 144 |
| B | 1 | 1 | 294 | 53 | 24 |
| B | 1 | 2 | 291 | 63 | 52 |
| B | 1 | 3 | 306 | 40 | 14 |
| B | 1 | 4 | 319 | 29 | 0 |
| B | 1 | 5 | 279 | 63 | 57 |
| B | 1 | 6 | 290 | 40 | 46 |
| B | 2 | 1 | 87 | 61 | 152 |
| B | 2 | 2 | 112 | 66 | 171 |
| B | 2 | 3 | 120 | 53 | 168 |
| B | 2 | 4 | 141 | 32 | 202 |
| B | 2 | 5 | 104 | 51 | 175 |
| B | 2 | 6 | 105 | 61 | 186 |
| B | 3 | 1 | 42 | 68 | 147 |
| B | 3 | 2 | 67 | 37 | 166 |
| B | 3 | 3 | 42 | 51 | 163 |
| B | 3 | 4 | 63 | 45 | 176 |
| B | 3 | 5 | 54 | 37 | 156 |
| B | 3 | 6 | 30 | 22 | 169 |
| B | 4 | 1 | 55 | 26 | 177 |
| B | 4 | 2 | 81 | 42 | 155 |
| B | 4 | 3 | 58 | 0 | 172 |
| B | 4 | 4 | 62 | 21 | 161 |
| B | 4 | 5 | 51 | 23 | 181 |
| B | 4 | 6 | 57 | 60 | 184 |
| C | 1 | 1 | 301 | 137 | 183 |
| C | 1 | 2 | 281 | 123 | 155 |
| C | 1 | 3 | 259 | 115 | 165 |
| C | 1 | 4 | 236 | 139 | 185 |
| C | 1 | 5 | 258 | 135 | 139 |
| C | 1 | 6 | 240 | 125 | 151 |
| C | 2 | 1 | 123 | 118 | 150 |
| C | 2 | 2 | 99 | 107 | 138 |
| C | 2 | 3 | 81 | 113 | 138 |
| C | 2 | 4 | 127 | 108 | 152 |
| C | 2 | 5 | 119 | 100 | 142 |
| C | 2 | 6 | 117 | 87 | 163 |
| C | 3 | 1 | 35 | 93 | 132 |
| C | 3 | 2 | 24 | 99 | 133 |
| C | 3 | 3 | 0 | 111 | 138 |
| C | 3 | 4 | 0 | 144 | 141 |
| C | 3 | 5 | 6 | 132 | 139 |
| C | 3 | 6 | 12 | 117 | 138 |
| C | 4 | 1 | 20 | 113 | 146 |
| C | 4 | 2 | 21 | 93 | 144 |
| C | 4 | 3 | 30 | 142 | 134 |
| C | 4 | 4 | 33 | 122 | 132 |
| C | 4 | 5 | 32 | 112 | 136 |
| C | 4 | 6 | 27 | 124 | 129 |
| D | 1 | 1 | 300 | 111 | 179 |
| D | 1 | 2 | 303 | 84 | 132 |
| D | 1 | 3 | 306 | 91 | 126 |
| D | 1 | 4 | 320 | 96 | 124 |
| D | 1 | 5 | 288 | 84 | 151 |
| D | 1 | 6 | 277 | 188 | 177 |
| D | 2 | 1 | 69 | 161 | 120 |
| D | 2 | 2 | 67 | 169 | 117 |
| D | 2 | 3 | 83 | 162 | 153 |
| D | 2 | 4 | 96 | 129 | 143 |
| D | 2 | 5 | 97 | 162 | 129 |
| D | 2 | 6 | 81 | 150 | 124 |
| D | 3 | 1 | 36 | 68 | 162 |
| D | 3 | 2 | 27 | 91 | 168 |
| D | 3 | 3 | 35 | 57 | 174 |
| D | 3 | 4 | 48 | 69 | 154 |
| D | 3 | 5 | 29 | 84 | 147 |
| D | 3 | 6 | 27 | 53 | 156 |
| D | 4 | 1 | 13 | 69 | 153 |
| D | 4 | 2 | 32 | 103 | 162 |
| D | 4 | 3 | 55 | 92 | 146 |
| D | 4 | 4 | 51 | 75 | 157 |
| D | 4 | 5 | 54 | 88 | 149 |
| D | 4 | 6 | 46 | 60 | 160 |
