# Supplementary material for: Agricultural Activities of a Meadow Eliminated Plant Litter from the Periphery of a Farmland in Inner Mongolia, China
Source: PLoS One. 2015 Aug 4;10(8):e0135077. doi: 10.1371/journal.pone.0135077 (PMC4524670; doi:10.1371/journal.pone.0135077)
Supplement: S7 Table — Score of Ax1 and Ax2 were used to draw a scatter plot (Fig 4). (DOCX) [file pone.0135077.s007.docx]

**S7 Table. Results of DCA analysis (Species).** Score of Ax1 and Ax2 were used to draw a scatter plot (**Figure 4**).

| Species | AX1 | AX2 | AX3 | N |
| --- | --- | --- | --- | --- |
| *Achnatherum sibiricum* (L.) Keng | -44 | 55 | 106 | 21 |
| *Adenophora crispata* (Korsh.) Kitag. | -113 | 197 | 265 | 2 |
| *Adenophora stenanthina* (Ledeb.) Kitag. | 133 | 63 | 192 | 17 |
| *Agropyron cristatum* (L.) Gaertn | 108 | -34 | 235 | 57 |
| *Allium anisopodium* Ledeb. | 34 | -137 | 171 | 9 |
| *Allium bidentatum* Fisch. Ex Prokh. | -24 | 35 | 164 | 34 |
| *Allium condensatum* Turcz. | 89 | -205 | 262 | 13 |
| *Allium senescens* L. | -46 | -52 | 239 | 21 |
| *Allium tenuissimum* L. | -12 | -3 | 195 | 37 |
| *Artemisia eriopoda* Bunge | -53 | 103 | 174 | 35 |
| *Artemisia frigida* Willd. | 0 | -13 | 115 | 36 |
| *Artemisia gmelinii* Web. ex Stechm. | 383 | 398 | 394 | 2 |
| *Artemisia pubescens* Ledeb. | 151 | -225 | 276 | 15 |
| *Artemisia sieversiana* Ehrhart ex Willd. | 353 | 137 | 291 | 21 |
| *Astragalus melilotoides* Pall. | -29 | 251 | 97 | 8 |
| *Bromus inermis* Leyss. | 383 | 10 | -37 | 28 |
| *Bupleurum scorzonerifolium* Willd. | -33 | 23 | 241 | 12 |
| *Carex korshinskyi* Kom. | 179 | 44 | 51 | 82 |
| *Chenopodium aristatum* L. | 303 | 189 | 270 | 34 |
| *Chenopodium glaucum* L. | 321 | 89 | 268 | 26 |
| *Cleistogenes squarrosa* (Trin.) Keng | -43 | -56 | 228 | 41 |
| *Clematis hexapetala* Pall. | 136 | 30 | 135 | 2 |
| *Cymbaria dahurica* L. | -12 | 194 | 70 | 46 |
| *Dianthus chinensis* L. | 46 | 248 | 49 | 16 |
| *Filifolium sibiricum* (L.) Kitam. | -50 | 225 | 79 | 39 |
| *Galium verum* L. | 204 | 225 | 123 | 25 |
| *Geranium sibricum* L. | 238 | 290 | 549 | 1 |
| *Heteropappus altaicus* (Willd.) Novopokr. | 6 | 35 | 139 | 9 |
| *Iris dichotoma* Pall. | -11 | 40 | 151 | 46 |
| *Iris ventricosa* Pall. | -56 | 186 | 96 | 24 |
| *Koeleria cristata* (L.) Pers. | 21 | 17 | 228 | 57 |
| *Lappula redowskii* (Horn) Greene | 253 | -19 | -208 | 1 |
| *Leontopodium leontpodioides* (Wild.) Beauv. | -55 | 49 | 211 | 11 |
| *Leymus chinensis* (Trin.) Tzvel. | 217 | 115 | 119 | 95 |
| *Linum perenne* L. | -85 | -46 | 229 | 2 |
| *Melilotoides ruthenica* (L.) Sojak | 143 | 210 | 106 | 45 |
| *Orostachys fimbriatus* (Turcz.) Berger | 35 | -377 | 56 | 1 |
| *Oxytropis myriophylla* (Pall.) DC. | -57 | 254 | 156 | 8 |
| *Poa subfastigiata* Trin. | 60 | -97 | 55 | 4 |
| *Polygonum divaricatum* L. | 238 | 290 | 549 | 21 |
| *Potentilla acaulis* L. | -48 | 44 | 141 | 1 |
| *Potentilla bifurca* L. | 170 | 180 | 125 | 32 |
| *Potentilla parvifolia* Fisch. ap. Lehm. | -76 | 307 | 38 | 46 |
| *Potentilla tanacetifolia*Willd. ex Schlecht. | -4 | 20 | 183 | 55 |
| *Potentilla verticillaris* Steph. ex Willd. | -19 | 158 | 170 | 48 |
| *Pulsatilla turczaninovii* Kryl. et Serg. | -29 | 95 | 138 | 43 |
| *Rumex* sp. | 101 | 455 | 282 | 1 |
| *Salsola collina* Pall. | 244 | 32 | 353 | 9 |
| *Sanguisorba officinalis* L. | 3 | 319 | 42 | 4 |
| *Saposhnikovia divaricata* (Turcz.) Schischk. | 94 | 164 | 209 | 56 |
| *Saussurea japonica* (Thunb.) DC. | 98 | 359 | -27 | 1 |
| *Scabiosa comosa* Fisch. ex Roem. et schult. | -21 | 279 | 142 | 20 |
| *Schizonepeta multifida* (L.) Briq. | -25 | 244 | 206 | 19 |
| *Scutellaria baicalensis* Georgi | 114 | 407 | 63 | 12 |
| *Scutellaria scordifolia* Fisch. ex Schrank | 96 | 207 | 44 | 18 |
| *Scutellaria* sp. | -41 | -59 | 232 | 14 |
| *Senecio kirilovii* Turcz. ex DC. | 7 | 190 | 58 | 17 |
| *Serratula centauroides* L. | 189 | 135 | 144 | 81 |
| *Setaria virdis* (L.) Beauv. | 330 | 209 | 307 | 31 |
| *Silene jenissconsis* Willd. | -65 | 294 | 68 | 19 |
| *Stellera chamaejasme* L. | -36 | 187 | 78 | 36 |
| *Stipa grandis* P. Smirn. | 3 | 63 | 180 | 66 |
| *Thalictrum petaloideum* L. | -3 | 177 | 119 | 40 |
| *Thalictrum squarrosum* Steph. ex Willd. | 186 | 49 | 254 | 23 |
| *Thermopsis lanceolata* R. Br. | 40 | 174 | 71 | 24 |
| unknown sp. 1 | -95 | 336 | 135 | 3 |
| unknown sp. 2 | -120 | 31 | 30 | 1 |
| Vicia amoena Fisch. | 190 | -60 | -4 | 14 |
